# Dual Extraction of Essential Oil and Podophyllotoxin from Creeping Juniper (*Juniperus horizontalis*)

**DOI:** 10.1371/journal.pone.0106057

**Published:** 2014-09-09

**Authors:** Charles L. Cantrell, Valtcho D. Zheljazkov, Camila R. Carvalho, Tess Astatkie, Ekaterina A. Jeliazkova, Luiz H. Rosa

**Affiliations:** 1 Natural Products Utilization Research Unit, Agricultural Research Service, United States Department of Agriculture, University, Mississippi, United States of America; 2 Sheridan Research and Extension Center, University of Wyoming, Sheridan, Wyoming, United States of America; 3 Laboratory of Systematic and Biomolecules of Fungi, Microbiology Department, Institute of Biological, Sciences Federal University of Minas Gerais, Belo Horizonte, Minas Gerais, Brazil; 4 Faculty of Agriculture, Dalhousie University, Truro, Nova Scotia, Canada; National Taiwan University, Taiwan

## Abstract

*Juniperus horizontalis* Moench (Family Cupressaceae), commonly called creeping juniper, is a widely distributed species in the United States and much of Canada. It is potentially a source for two important chemical products, the anticancer drug synthetic precursor, podophyllotoxin and essential oils. The objectives of this study were to ascertain the likelihood of utilizing *J. horizontalis* needles for the simultaneous production of both (−)-podophyllotoxin and essential oil components and to determine the optimum distillation time (DT) needed for the production of essential oil containing a specific ratio of constituents. Eleven different distillation times were tested in this study: 20, 40, 80, 160, 180, 240, 480, 600, 720, 840, and 960 min. Total essential oil content increased with increasing distillation time from a minimum of 0.023% at 20 min to a maximum of 1.098% at 960 min. The major constituents present in the oil were alpha-pinene, sabinene, and limonene. The percent concentration of sabinene in the essential oil varied from a high of 46.6% at 80 min to a low of 30.2% at 960 min, that of limonene changed very little as a result of distillation time and remained near 30% for all distillation times, whereas the concentration of alpha-pinene was 9.6% at 20 min DT and decreased to 4.2% at 960 min. Post distillation analysis of needles revealed elevated amounts of (−)-podophyllotoxin remaining in the tissue varied in the amount of podophyllotoxin present, from a low of 0.281% to a high of 0.364% as compared to undistilled needles which gave 0.217% podophyllotoxin. As a result of this study, specific essential oil components can now be targeted in *J. horizontalis* by varying the distillation time. Furthermore, needles can be successfully utilized as a source of both essential oil and podophyllotoxin, consecutively.

## Introduction

*Juniperus* species are cultivated worldwide and commonly found from sea level to above timberline. In many parts of North America *Juniperus* species have become almost weedy in nature and have invaded millions of acres of rangeland and farms [1]. The genus *Juniperus* consists of 67 species and recent reports have identified *Juniperus horizontalis* Moench as a possible source for the anticancer drug precursor, podophyllotoxin [2], [3]. Creeping juniper (*J. horizontalis*) is commonly grown as an ornamental and its oil is useful in apothecary, fragrance, and pharmaceutical industries.

*Podophyllum* species such as the Himalayan mayapple (*Podophyllum hexandrum* Royle) are sources of (−)-podophyllotoxin, a lignan useful in the semi-synthesis of commercially used cancer treating drugs such as etoposide and teniposide [2], [4], [5], [6]. These compounds have been used for the treatment of lung cancer, testicular cancer, neuroblastoma, hepatoma, and other tumors [7], [8]. *P. hexandrum* is reportedly intensively collected and utilized for bulk extraction and production of (−)-podophyllotoxin. Some reports have suggested it may become endangered due to overharvesting as the demand for (−)-podophyllotoxin derived drugs continues to increase [9]. Despite the reports on progress and improvements towards the total synthesis of (−)-podophyllotoxin, many fall short at becoming economically feasible in a commercial process. A more viable alternative domestic source of (−)-podophyllotoxin seems to be *Juniperus* species.

Essential oils and cedarwood oils from *Juniperus* species, primarily *J. virginiana*, are used in the fragrance and flavor industry [10]. While limited research has been done on extracts of the needles of *J. horizontalis* [11], [12], [13], the essential oil obtained from the wood of the plant (cedarwood oil) is used in a broad range of products and known for unique properties, such as aroma and toxicity that repel and kill many pests [14].

Previously, it was shown that podophyllotoxin does not degrade during a 90-min steam distillation of *J. virginiana* [15] and hence it may be possible to obtain intact podophyllotoxin following steam distillation. It was also reported that for maximum essential oil yield, female *J. scopulorum* needed to be distilled for at least 240 min [16], whereas male *J. scopulorum* needed to be distilled for 840 min [17]. Previous authors reported essential oil composition of *J. horizontalis*, however, the biomass was extracted for 90 min [2], or 120 min [11], [12], [13]. These shorter durations of DT may not have extracted the total oil, and secondly, the oil composition might have been different depending on the duration of the distillation time (DT).

The hypothesis of this study was that the duration of distillation time may affect essential oil yield and composition of *J. horizontalis*, and that podophyllotoxin may not degrade during extended distillation times of 12 or more hours. Furthermore, the duration of the distillation time may be used to obtain essential oil with specific chemical profile. Therefore, the objectives of this study were: (1) to ascertain the likelihood of utilizing *J. horizontalis* needles for the simultaneous production of both (−)-podophyllotoxin and essential oil components and (2) to determine the optimum distillation time needed for the production of essential oil containing the lowest/highest purity possible for a particular constituent(s).

## Materials and Methods

### 2.1. Plant material and growing conditions

The plant material used in this study was collected in the Big Horn Mountains on 5 December, 2012. The plant material was obtained from a single creeping juniper plant, #131, which in a previous study, was identified to have relatively high concentration of podophyllotoxin [18]. This specific plant was found at elevation of 2,070 m above the sea level, with GPS coordinates N 440 37.108′ W 1070 05.072′. This juniper was identified as creeping juniper by Ms. Bonnie Heidel, a botanist at the Wyoming Natural Diversity Database, University of Wyoming [18]. Permission for sampling of junipers in the Big Horn Mountains National Forest was issued to Dr. Valtcho Jeliazkov by Mr. Clarke McClung, Tongue District Ranger on March 7, 2012 {authorization ID: TNG551, from U.S. Department of Agriculture, Forest Service, Temporary Special use permit with expiration date 12/31/2012 (FSH 2709.11 sec.54.6, Authority Organic Administration Act June 4, 1897)}.

### 2.2. Essential oil extraction

The essential oils were extracted via steam distillation, in 2-L steam distillation units as previously described [15], [19]. Fresh samples (500 g) consisted of leaves (needles) and smaller than 2 mm in diameter branches. Prior to distillation, the samples were chopped into 2.5 cm long pieces. There were 11 different distillation times tested in this study as follows: 20, 40, 80, 160, 180, 240, 480, 600, 720, 840, and 960 min. These distillation times were selected based on previous trials with Rocky Mountain juniper, *J. socpulorum* [17]. The times were measured from the beginning of the distillation (when the first drop of essential oil was deposited in the Florentine); at the end of the distillation time the heating was cut off, the steam removed, the Florentine was also removed and the essential oil decanted. Each essential oil sample was weighed on analytical scale and kept in a freezer at minus 5°C until the gas chromatography analyses can be performed.

### 2.3. Gas chromatography-FID analysis of the essential oils

The essential oil samples of creeping juniper (all samples in three replicates) were analyzed on a gas chromatograph (GC, Hewlett Packard model 6890, Hewlett-Packard, Palo Alto, CA, USA). The carrier gas was helium at a flow rate of 40 cm/sec, 11.7 psi (60°C), 2.5 ml/min constant flow rate. The injection was split 60∶1, 0.5 µL, the injector temperature was 220°C. The GC oven temperature program was as follows: 60°C for 1 min, 10°C/min to 250°C. The column was HP-INNOWAX (cross-linked PEG; 30 m × 0.32 mm × 0.5 µm), and the flame ionization detector (FID) temperature was set to 275°C. Individual constituents of the creeping juniper essential oils are expressed as percentage of the total oil. The identification of individual constituent peaks was done with the use of internal standards, by retention time and by mass-spectroscopy.

### 2.4 Quantitative analysis of podophyllotoxin

Podophyllotoxin analysis was performed essentially as described previously [2], [20]. Briefly, 40 mg of each dry tissue sample was incubated at 20°C with 0.6 mL of 25 mM potassium phosphate buffer (pH of 7.0) on an Eppendorf Thermomixer R for 30 min at 750 rpm. Subsequently, 0.6 mL of ethyl acetate was added, and the incubation continued for an additional 5 min in the same manner. The aqueous and organic partitions were separated by centrifuge (Savant speed vac, svc 200). The organic layer was removed using a Pasteur pipette and evaporated under a stream of N_2_, leaving the organic soluble material to be dissolved in methanol (100%) and analyzed by HPLC. Extracts were analyzed using an HPLC system (Agilent 1100 series consisting of a vacuum degasser, quaternary pump, ALS autosampler, a diode array detector, and an Agilent Eclipse XDB-C18, 4.6 mm × 150 mm, 5 µm column). The injection volume for all samples and for the podophyllotoxin standard was 10 µL and standards and samples were analyzed at 21°C. An analytical isocratic method was used (28% acetonitrile: 72% deionized water with 0.1% TFA) for 20 min followed by a 5 min column wash with methanol and a 10 minute re-equilibration. Analytes were detected at 220 nm.

Podophyllotoxin was purchased from Sigma-Aldrich (St. Louis, MO). Individual concentration gradients were prepared for podophyllotoxin to obtain a standard curve using five concentration points imposed by using response factors and regression coefficients independently. Response factors were calculated using the equation RF  =  DR/C, where DR was the detector response in peak area (PA) and C was the podophyllotoxin concentration. Confirmed integrated peaks were then used to determine the percentage of podophyllotoxin in the extract. The RF of the target chemical constituent was used to determine the “percent” for each sample using the equation: PA/RF/C × 100 = % (peak area/response factor/concentration) in the plant tissue.

### 2.5. Statistical analysis

The effect of distillation time on essential oil content, and the concentration of alpha-thujene, alpha-pinene, sabinene, myrcene, alpha-terpinene, limonene, gamma-terpinene, terpinolene, 4-terpineol, pregeijerene B, delta-cadinene, elemol, and podophyllotoxin was determined using a one-way analysis of variance. For each response, the validity of model assumptions was verified by examining the residuals as described in Montgomery [21]. Since the effect of distillation time was significant (p-value < 0.05) on all responses, multiple means comparison was completed using Duncan's multiple range test at the 5% level of significance, and letter groupings were generated. The analysis was completed using the GLM Procedure of SAS [22].

The relationship between distillation time and essential oil content, and the concentration of alpha-thujene, alpha-terpinene, gamma-terpinene, terpinolene, delta-cadinene, elemol, was adequately described by the Power – concave (Eq. 1, with 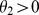), the relationship between distillation time and the concentration of alpha-pinene was adequately described by the Power - convex (Eq. 1, with 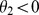), and that between distillation time and the concentration of sabinene, myrcene, limonene, 4-terpineol, and pregeijerene B was described by either a second order (Eq. 2) or a third order (Eq. 3) polynomial. While the Power model is nonlinear, the second and third order polynomial models are linear. The parameters of the nonlinear model were estimated iteratively using the NLIN Procedure of SAS [22].
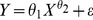
(Eq. 1)

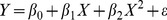
(Eq. 2)

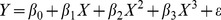
(Eq. 3)


Where Y is the dependent (response) variable, X is the independent (distillation time) variable, and the error term ε is assumed to have normal distribution with constant variance.

## Results and Discussion

**Figure 1:**
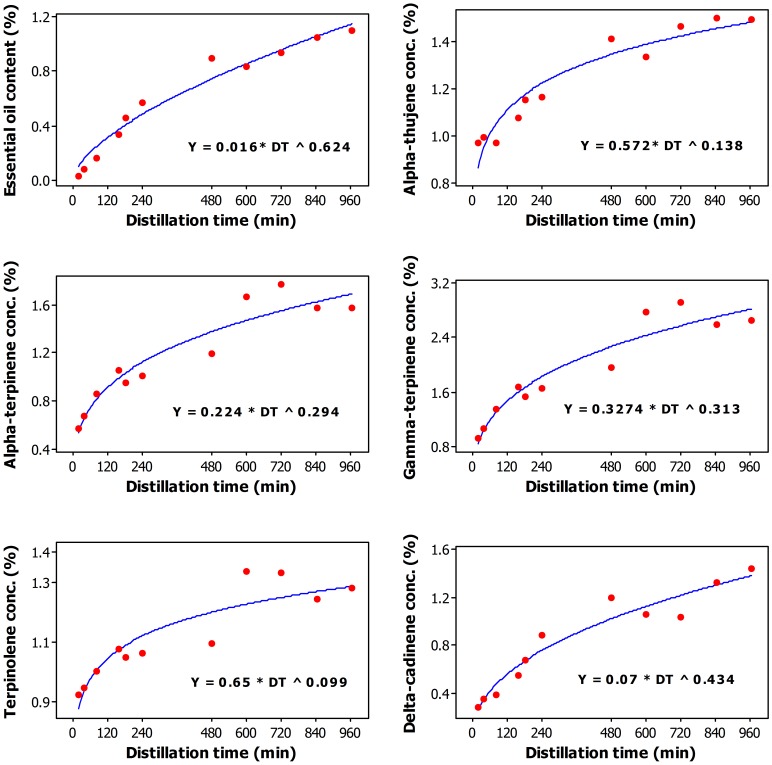
Plot of Distillation time versus essential oil content (wt/wt%) and the concentration in essential oil (%) of alpha-thujene, alpha-terpinene, gamma-terpinene, terpinolene, and delta-cadinene along with the fitted (solid line) Power–concave regression model.

Total essential oil content increased with increasing distillation time (Table 1) from a minimum of 0.023% at 20 min to a maximum of 1.098% at 960 min. A plot of distillation time versus essential oil content together with a fitted Power-concave model that described the relationship very well is shown in Figure 1. A steady and significant increase was observed from 20 min to 840 min. Overall, the effect of distillation time relative to each individual constituent was significant.

**Table 1:** Mean essential oil content (%)^b^, and the concentrations (%)^c^ of alpha-thujene, alpha-pinene, sabinene, myrcene, alpha-terpinene, limonene, gamma-terpinene, terpinolene, 4-terpineol, pregeijerene B, delta-cadinene, elemol, in essential oil obtained from the 11 distillation times, and podophyllotoxin content (%)^d^ in extracted or unextracted samples.

DT (min)	Essent. oil content	Alpha-thujene	Alpha-pinene	Sabinene	Myrcene	Alpha-terpinene	Limonene	Gamma-terpinene	Terpinolene (%)	4-Terpineol	Pregeijerene B	Delta-cadinene	Elemol	Podophyllotoxin
	------------------------------------------------------------------------------%^a^ -------------------------------------------------------------------------------------------------------------------------													
20	0.023 i	0.97 d	9.62 a	44.2 b	2.23 cd	0.57 f	29.6 abc	0.91 h	0.923 f	0.83 e	1.60 c	0.273 g	0.51 f	0.363 a
40	0.082 h	0.99 d	7.42 b	45.5 ab	2.30 abcd	0.68 f	29.5 bc	1.06 gh	0.943 ef	1.11 de	1.91 c	0.350 g	0.72 f	0.364 a
80	0.162 g	0.97 d	4.69 c	46.6 a	2.30 abcd	0.86 e	29.8 abc	1.35 fg	1.000 de	1.52 cd	2.40 b	0.387 g	1.31 e	0.344 abc
160	0.329 f	1.07 cd	4.31 c	43.8 bc	2.39 ab	1.05 d	30.2 abc	1.68 e	1.073 c	1.98 ab	2.62 ab	0.547 f	2.45 d	0.336abcd
180	0.460 e	1.15 c	4.41 c	44.5 b	2.41 a	0.95 de	30.5 ab	1.54 ef	1.047 cd	1.78 bc	2.65 ab	0.677 e	2.22 d	0.356 a
240	0.566 d	1.16 c	4.40 c	42.0 c	2.35 abc	1.01 d	30.6 a	1.64 e	1.060 cd	1.79 bc	2.78 a	0.883 d	3.40 c	0.353 ab
480	0.892 b	1.41 ab	4.31 c	37.9 d	2.22 cd	1.20 c	30.5 ab	1.96 d	1.093 c	1.76 bc	2.57 ab	1.200 b	5.56 b	0.329abcd
600	0.830 c	1.33 b	4.10 c	32.2 e	2.26 bcd	1.66 ab	30.3 ab	2.78 ab	1.333 a	2.38 a	2.63 ab	1.060 c	7.87 a	0.298 bcd
720	0.939 b	1.46 a	4.40 c	31.6 ef	2.22 cd	1.76 a	29.9 abc	2.91 a	1.330 a	2.22 a	2.48 ab	1.027 c	8.40 a	0.281 d
840	1.045 a	1.49 a	4.54 c	31.8 ef	2.20 d	1.57 b	30.0 abc	2.59 c	1.243 b	1.95 ab	2.48 ab	1.317 b	8.23 a	0.325abcd
960	1.098 a	1.49 a	4.19 c	30.2 f	2.19 d	1.58 b	29.1 c	2.66 bc	1.277 ab	1.71 bc	2.57 ab	1.440 a	9.41 a	0.290 cd
Unextracted														0.217 e

a Within each column, means followed by the same letter are not significantly different at the 5% level of significance.

b Percentage of essential oil by weight in fresh plant material.

c Percentage of each analyte in essential oil by weight.

d Percentage of podophyllotoxin by weight in dry needles.

**Figure 2:**
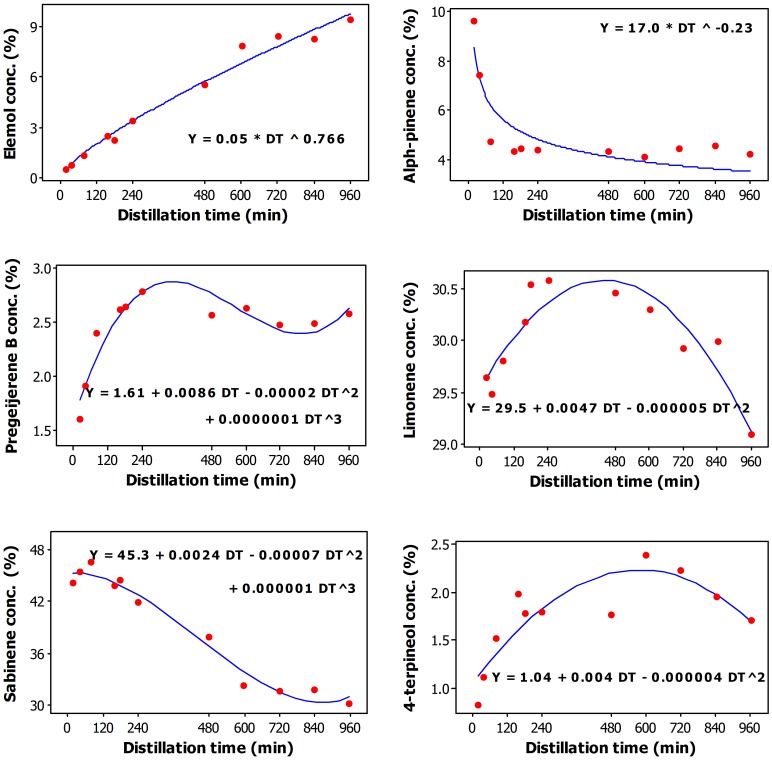
Plot of Distillation time versus the content (wt/wt%) in essential oil of elemol, alpha-pinene, pregeijerene B, limonene, sabinene, and 4-terpineol along with the fitted (solid line) Power–concave (elemol), Power-convex (alpha-pinene), third order polynomial (pregeijerene B, sabinene), and Second order polynomial (limonene, 4-terpineol) regression models.

The percent concentration of alpha-thujene in the essential oil was also adequately described by a Power-concave model (Figure 1) and increased from around 1% at 20–160 min to a maximum of 1.5% at 720 min; further increase of the duration of the DT to 960 min did not affect the concentration of this oil constituent (Table 1). Similarly, alpha-terpinene (Figure 1), gamma-terpinene (Figure 1), terpinolene (Figure 1), delta-cadinene (Figure 1), elemol (Figure 2), all can be adequately described by the Power-concave model.

The percent concentration of alpha-terpinene in the essential oil increased from a low of 0.57% at 20 min to a maximum of 1.76% at 720 min (Table 1). The percent concentration of limonene in the essential oil changed very little as a result of distillation time (Table 1) and remained near 30% for all distillation times. The percent concentration of gamma-terpinene in the essential oil increased from a low of 0.91% at 20 min to a maximum of 2.91% at 720 min (Table 1). Terpinolene percent concentration in the essential oil increased from a low of 0.923% at 20 min to a maximum of 1.333% at 600 min (Table 1). Delta-cadinene percent concentration in the essential oil increased from a low of 0.273% at 20 min to a maximum of 1.440% at 960 min (Table 1). Elemol percent concentration in the essential oil increased from a low of 0.51% at 20 min to a maximum of 9.41% at 960 min (Table 1).

The percent concentration of alpha-pinene in the essential oil can be adequately described by a Power-convex model (Figure 2) and decreased from a high of 9.62% at 20 min to a 4.7% at 80 min; further increase of DT did not significantly change the concentration of this constituent (Table 1). Alpha-pinene was the only constituent to follow a declining (convex) pattern.

The percent concentration of sabinene in the essential oil can be adequately described by a third order polynomial model (Figure 2) and varied from a high of 46.6% at 80 min to a low of 30.2% at 960 min (Table 1). Pregeijerene B was the only other constituent to adequately follow a third order polynomial model. Pregeijerene B percent concentration in the essential oil varied from a high of 2.78% at 240 min to a low of 1.60% at 20 min (Table 1).

Limonene (Figure 2) and 4-terpineol (Figure 2) can be adequately described by a second order polynomial model. Limonene percent concentration in the essential oil varied from a high of 30.6% at 240 min to a low of 29.5% at 40 min (Table 1). 4-terpineol percent concentration in the essential oil varied from a low of 0.83% at 20 min to a high of 2.38% at 600 min (Table 1).

The essential oil composition of the *J. horizontalis* in this study was dissimilar to the one reported previously [2], in which the plant material was distilled for 90 min. For example, the major oil constituents at 80 min DT in the present study were 4.7% alpha-pinene, 46.6% sabinene, 2.3% myrcene, 29.8% limonene, while the oil constituents in *J. horizontalis* in [2] were 0.47% beta-pinene, 1.82% myrcene, and 36.6% limonene. These differences might be due to different genetic material used in the two studies: wild collected *J. horizontalis* from the Big Horn Mountains in Wyoming was used in this study, whereas the *J. horizontalis* in [2] was the ornamental variety ‘Plumosa compacta’.

Furthermore, reported [12] essential oil composition of *J. horizontalis* extracted for 120 min, contained 1.7% alpha-pinene, 37.2% sabinene, 2.8% myrcene, 3.5% limonene, 3.9% terpinen-4-ol, and was comparable to the essential oil composition obtained at 80 or 160 min DT in the present study (Table 1). The *J. horizontalis* analyzed by [12] was collected along the Saskatchewan River in Saskatoon, Canada (52.1333° N, 106.6833° W), which is approximately at the same western longitude as the Big Horn Mountains in Wyoming, USA (N 44°37.108′ N, W 107°05.072′).

Our study demonstrated the need for longer duration of the DT in order to extract the total oil from *J. horizontalis*. A study [11] reported 0.33 and 0.38% oil content in juvenile and adult leaves of *J. horizontalis* extracted for 2 hours, which corresponds to the 0.33% oil content at 160 min DT in our study. However, our study showed that the actual total oil content of *J. horizontalis* when extracted for 840 or 960 min was over 1.0%, which is 3 times higher than the one reported in previous studies [11], [2].

Podophyllotoxin quantitative analysis was performed essentially as described previously [2], [20] using HPLC methods. *J. horizontalis* needles that were not extracted using steam distillation gave 0.217% podophyllotoxin. The amount of podophyllotoxin in needles that had been used for steam distillation varied from a low of 0.281% at a 720 min distillation to a high of 0.364% for a 40 min distillation. Interestingly, the concentration of podophyllotoxin in the unextracted samples was significantly less than its concentration in the extracted samples from all DT. Perhaps this can be explained by the fact that much of the podophyllotoxin that exists in the plant is likely in the form of a glycoside and not an aglycone. The distillation may be converting much of the glycoside into its corresponding aglycone (and sugar) by hydrolysis. Canel et al. reported on this conversion; however, more research is needed to address this phenomenon [20].

Although collected from the same plant, podophylloxin concentration in this study was lower than the previously reported 0.457% [18]. These differences were most probably due to the different sampling time and seasonal variations in podophyllotoxin concentrations: the samples in [18] were collected in March 2012, while the samples in this study were collected in Dec, 2012. The podophylotoxin concentration in *J. horizontalis* in this study was higher than previously reported podophylotoxin concentration for two *J. horizontalis* ornamental varieties ‘Wiltonii’ (0.138%) and ‘Plumosa Compacta’ (0.351%) [2]. The results from this and previous studies indicate podophylotoxin may vary significantly due to the genetic material /collection site and collection time.

## Conclusions

As a result of this study, it has been demonstrated that *J. horizontalis* needles can be successfully utilized as a source of essential oil and podophyllotoxin simultaneously. It was demonstrated that the total essential oil content of *J. horizontalis* was over 1%, which is approximately 3 times higher than that reported previously. In order to obtain the total amount of oil, *J. horizontalis* biomass must be steam distilled for at least 840 min. It has also been shown that podophyllotoxin yield from needles may increase as a result of steam distillation. The length of steam distillation did not dramatically affect the podophyllotoxin yield. Furthermore, specific essential oil components can now be targeted in *J. horizontalis* by varying the DT, or an essential oil with a desirable composition can be generated. The findings of this study demonstrated the need for reporting the duration of DT when reporting oil content and composition of *J. horizontalis*.
